# Calendar

**DOI:** 10.1155/S1463924684000109

**Published:** 1984


					Journal of Automatic Chemistry, Volume 6, Number (January-March 1984), page 46
Calendar
Editor's note
Organizers of conferences, seminars, etc.
should send details for inclusion in the
calendar as soon as the relevant infor-
mation is available and not later than three
months before the event.
February 1984
Automation in the Cosmetics Industry
To be held on 15 February 1984 in
Northampton, UK.
Further information from C. J. Jackson,
Occupational Hygiene Laboratory, 403
Edgware Road, London NW2 6LN.
The Place of Micro and Trace Analysis in
Present-Day Teaching
To be held on 15 February in London.
Further information from the Analytical
Division, Royal Society of Chemistry,
Burlington House, London W1 V OBN.
Statistics in Analytical Chemistry
To be held on 14 March in Lancaster,
UK.
Further information from Miss P. E.
Hutchinson (see above).
April 1984
New Concepts in Quality Control
To be held on 2 and 5 April 1984 in
Cambridge, UK.
Further information from C. Y. Jackson
(see above).
Analytica 84
To be held from 10 to 13 April 1984 in
Mtinchen, FR Germany.
Further information from Dr Rosmarie
Vogel, Abteilungfiir Klinische Chemie und
Klinische Biochemie in der Chirurgischen
Klinik Innenstadt der Universitgtt
Miinchen, Nussbaumstrasse 20, D 8000
Miinchen 2, FR Germany.
16th Annual Symposium on Advanced
Analytical Concepts for the Clinical
Laboratory
To be held on 12 and 13 April 1984 at the
Hilton Hotel in Knoxville, Tennessee,
USA.
Further information from Debbie
Woodcock, Meetings Co-ordinator,
AACC, 1725 K. Street, Washington, D.C.
20006, USA.
Recent Developments and Applications in
Ion Chromatography
To be held on 22 February in
Warrington, UK.
Further information from Miss P. E.
Hutchinson, Analytical Division, Royal
Society of Chemistry, Burlington House,
London W1 V OBN.
Advances in Chromatography:
Pharmaceutical and Clinical Applications
To be held on 28 and 29 February in
Edinburgh, UK.
Further information from Miss P. E.
Hutchinson (see above).
Royal Society of Chemistry: Annual
Chemical Congress
To be held from 16 to 19 April 1984 at the
University of Exeter, UK.
Further information from Dr John F.
Gibson, The Royal Society of Chemistry,
Burlington House, London W1 V OBN.
Xllth International Congress of Clinical
Chemistry
To be held from 29 April to 4 May 1984 in
Rio de Janeiro, Brazil.
Further information from the Executive
Secretary, XIIth International Congress,
Rua Vicente Licinio 95, Cep 20270, Rio de
Janeiro, Brazil.
March 1984
35th Pittsburgh Conference and
Exposition
To be held from 5 to 10 March 1984 in
Atlantic City, New Jersey, USA.
Further information from Mrs Linda
Briggs, Pittsburgh Conference, Depart-
ment J-212, 437 Donald Road, Pittsburgh,
Pennsylvania 15235, USA.
Automated Clinical Analysis
To be held on 7 March 1984 at Thames
Polytechnic in London.
Further information from C. J. Jackson
(see above).
May 1984
Principles ofAutomation and Applications
of Robotics
To be held on 9 and 10 May 1984 at the
Scientific Societies Lecture Theatre,
23 Savile Row, London.
Further information from C. J. Jackson
(see above).
June 1984
2nd International Conference on
Chromatography and Mass Spectrometry
in Biomedical Sciences
To be held from 18-20 June 1984 in
Milan, Italy.
46
Further informationfrom Alberto Frigerio,
Italian Group for Mass Spectrometry in
Biochemistry and Medicine, Via Eustachi
36, 20129 Milan, Italy.
Quality Control of Packaging: Food,
Beverages, Pharmaceuticals and Cos-
meties
To be held from 20 to 22 June 1984 in
St. Andrews, UK.
Further information from C. J. Jackson
(see above).
September 1984
Analyticon 84
To be held from 4 to 6 September in
London.
Further information from Gambica, 8
Leicester Street, London WC2H 7BN.
September 1984
Third European Symposium on Thermal
Analysis and Calorimetry
To be held from 9 to 15 September 1984 at
the Congress-Center-Casino Interlaken
in Interlaken, Switzerland.
Further information from ESTAC 3 84,
Dr E. Marti, Im Langen Lob 181,
CH 40.54 Basel, Switzerland.
October 1984
2nd International Congress on
Automation and New Technology in the
Clinical Laboratory
To be held from 15 to 18 October 1984 in
Barcelona, Spain.
For further information contact 2nd
International Congress on Automation and
New Technology in the Clinical
Laboratory, IV Congreso Nacional de la
Sociedad Espahola de Quimica Clinica,
Apartado de Correos 543, Barcelona,
Spain.
November 1984
14th Annual Symposium on the Analytical
Chemistry of Pollutants and the 3rd
International Congress on Analytical
Techniques in Environmental Chemistry
To be held from 22-24 November 1984 at
the Palacio de Congresos in Barcelona,
Spain.
For further information contact 14th
Annual Symposium--3rd International
Congress--Expoquimia, Avda. Reina Ma.
Christina, Barcelona 4, Spain.
1985 MEETINGS
IUPAC 1985
To be held from 9 to 13 September 1985 in
Manchester, UK.
Further information fi'om The Royal
Society of Chemistry, Burlington House,
London W1 V OBN.

				

